# 9-*cis*-Retinoic Acid and Troglitazone Impacts Cellular Adhesion, Proliferation, and Integrin Expression in K562 Cells

**DOI:** 10.1371/journal.pone.0093005

**Published:** 2014-03-26

**Authors:** Amanda M. Hanson, Jessica Gambill, Venusa Phomakay, C. Tyler Staten, Melissa D. Kelley

**Affiliations:** 1 Department of Chemistry and Biochemistry, University of Arizona, Tucson, Arizona, United States of America; 2 College of Medicine, University of Arkansas Medical Sciences, Little Rock, Arkansas, United States of America; 3 College of Pharmacy, Harding University, Searcy, Arkansas United States of America; 4 Department of Chemistry, University of Central Arkansas, Conway, Arkansas, United States of America; Thomas Jefferson University, United States of America

## Abstract

Retinoids are established pleiotropic regulators of both adaptive and innate immune responses. Recently, troglitazone, a PPAR gamma agonist, has been demonstrated to have anti-inflammatory effects. Separately, retinoids and troglitazone are implicated in immune related processes; however, their combinatory role in cellular adhesion and proliferation has not been well established. In this study, the effect of 9-*cis*-retinoic acid (9-*cis*-RA) and troglitazone on K562 cellular adhesion and proliferation was investigated. Troglitazone exposure decreased K562 cellular adhesion to RGD containing extracellular matrix proteins fibronectin, FN-120, and vitronectin in a concentration and time-dependent manner. In the presence of troglitazone, 9-*cis*-retinoic acid restores cellular adhesion to levels comparable to vehicle treatment alone on fibronectin, FN-120, and vitronectin substrates within 72 hours. Due to the prominent role of integrins in attachment to extracellular matrix proteins, we evaluated the level of integrin α5 subunit expression. Troglitazone treatment results in decrease in α5 subunit expression on the cell surface. In the presence of both agonists, cell surface α5 subunit expression was restored to levels comparable to vehicle treatment alone. Additionally, troglitazone and 9-*cis*-RA mediated cell adhesion was decreased in the presence of a function blocking integrin alpha 5 inhibitor. Further, through retinoid metabolic profiling and HPLC analysis, our study demonstrates that troglitazone augments retinoid availability in K562 cells. Finally, we demonstrate that troglitazone and 9-*cis*-retinoic acid synergistically dampen cellular proliferation in K562 cells. Our study is the first to report that the combination of troglitazone and 9-*cis*-retinoic acid restores cellular adhesion, alters retinoid availability, impacts integrin expression, and dampens cellular proliferation in K562 cells.

## Introduction

The literature is rich with the influence of vitamin A (retinol) and its analogs, retinoids, on establishing and maintaining proper immunity [1], [2], [3], [4], [5], [6], [7], [8], [9], [10]. Retinol was coined as an “anti-infective vitamin” as early as the 1920’s [11], [12], [13]. Adaptive and innate immune responses are regulated by vitamin A [14]. More recently, biologically active metabolites of retinol, all-*trans*-retinoic acid and 9-*cis*-retinoic acid, have been reported to mediate immunological functions such as differentiation, proliferation, and transmigration of leukocytes [4], [15], [16], [17], [18], [19].

All-*trans*-retinoic acid (*t*-RA) and 9-*cis*-retinoic acid (9-*cis*-RA) activate retinoic acid receptors (RAR α, β, and γ) and retinoid X receptors (RXR α, β, and γ) [20], [21], [22], [23], [24]. These retinoids act as ligand-dependent transcription factors with *t*-RA activating RARs, while 9-*cis*-RA serves as a pan-agonist for RARs and RXRs. These receptor complexes act as heterodimers or homodimers binding to specific retinoid response elements, RARE and RXRE, in the promoter of target genes [25]. RXRs are promiscuous receptors forming heterodimers with other ligand-dependent members of the nuclear receptor family including thyroid hormone, vitamin D receptors, and more recently peroxisome proliferator activated receptor gamma (PPARγ) [2], [26].

PPAR gamma has been implicated in a variety of pathologies most notably, diabetes and obesity. PPARγ agonists, such as those belonging to the thiazolidinedione class (troglitazone, roglitazone, and pioglitazone) have been used in the treatment of hyperlipidaemia and hyperglycemia. Recently, the role of PPARγ in regulation of inflammation and immune response has become more of a focus. PPARγ agonist activation generally results in anti-inflammatory effects including decreases in macrophage inflammatory cytokines, apoptosis of macrophages, and inhibition of activated T-cell proliferation [27], [28], [29], [30]. Specifically, PPARγ-RXRα pairing has resulted in agonist-induced mitogen-activated protein kinase activation and inhibition of nuclear factor-kappa B [31], [32], [33].

Cellular abundance of *t*-RA and 9-*cis*-RA is directly controlled through a variety of processes including oxidative metabolism and sequestration [2]. The cytochrome P_450_ (CYP) family of monooxygenases are involved in the biotransformation of variety of endogenous and exogenous compounds including *t*-RA and 9-*cis*-RA. The predominant pathway is oxidation at the 4-position of the cyclohexenyl ring to form 4-hydroxy- and 4-oxo-retinoic acid or 4-hydroxy and 4-oxo-9-*cis*-retinoic acid [34], [35]. Several human CYP isoforms are capable of metabolizing *t*-RA including CYP2C8, CYP3A4, CYP2C9, and more recently CYP26 [36], [37], [38]. Interestingly, the PPARγ agonist, troglitazone is a strong inhibitor of CYP2C8 and CYP3A4 [39]. Ultimately, retinoid availability dictates retinoid receptor heterodimer/homodimer pairings, effectively regulating transcription of retinoid responsive genes that are involved in mediating cellular adhesion and proliferation.

Cell surface adhesion molecules, such as integrins, mediate cell-cell and cell-matrix interactions and are essential for maintaining cell homeostasis. Integrins, a family of transmembrane heterodimeric receptors consisting of non-covalently linked α and β subunits, are considered to be the principle receptors involved in attachment to the extracellular matrix. Integrin engagement with their counter ligands leads to signaling processes that are critical for cellular proliferation and migration [40], [41], [42], [43], [44]. The α5β1 integrin subclass is involved in the remodeling of the lymphatics during inflammation [45]. The α5β1 integrins are fibronectin receptors with the arg-gly-asp (RGD) sequence in the FNIII domain 10 being the crucial attachment site for α5β1 [46]. Interestingly, both retinoids and thiazolidinediones have been implicated in altering integrins and their counter-receptors. Retinoids influence expression of classical integrin ligands, including VCAM-1 and fibronectin, and modulate integrin expression, including αvβ3, αvβ5, α4β1, and α4β7 [47], [48], [49], [50], [51]. Troglitazone reduces VCAM-1 expression and adhesion to α4β7 integrins in the presence of TNF-α [10], [52].

Although retinoids and thiazolidinediones have been independently investigated with respect to their roles in cellular adhesion and proliferation, there is a shortage of information evaluating the combination of these agonists on these crucial immunological functions. In this study, we evaluated the combinatory treatment of troglitazone and 9-*cis*-RA on cellular adhesion and proliferation in the human erythroleukemia cell line, K562. We present evidence that troglitazone dampens cellular adhesion to RGD containing extracellular matrix proteins in a time and concentration-dependent manner, and cellular adhesion is restored to these ligands by 9-*cis*-RA. Additionally, through retinoid metabolic profiling, we demonstrate that troglitazone increases the bioavailability of 9-*cis*-RA. Further, we investigated the effects of these agonists on the integrin α5 subunit cell surface expression. Our study is the first to demonstrate that the combinatory treatment of the PPAR agonist, troglitazone, and the RXR agonist, 9-*cis*-RA, results in augmented cellular adhesion, modulated retinoid metabolism, restored integrin expression, and dampened cellular proliferation in a human erythromyeblastoid leukemia cell line.

## Materials and Methods

### Reagents and Chemicals

Human fibronectin was purchased from BD Biosciences (Bedford, MA). The purified human fibronectin alpha-chymotryptic fragment, FN-120, and the Fibronectin 40 kDa α Chymotryptic Fragment (Heparin-binding region), FN-40, was purchased from Millipore (Temecula, CA). Purified human vitronectin was purchased from R&D systems (Minneapolis, MN). The function blocking antibodies SAM-1 (anti-integrin α5), B-D15 (anti-integrin β1), F11 (anti-integrin β3), MOPC-21 (isotype control), and the secondary goat anti-human IgG FITC conjugated antibody were purchased from Abcam (Cambridge, MA). Anti-integrin αvβ3 and αvβ5 FITC conjugated antibodies were purchase from Millipore. The anti α8 integrin and the secondary rat anti-mouse IgG_2B_ FITC conjugated antibody was purchased from R&D systems. 9-*cis*-retinoic acid, 13-*cis*-retinoic acid, troglitazone, and *p*-nitro-phenyl phosphate were purchased from Sigma (St. Louis, MO). 4-oxo-retinoic acid was kindly provided by Hoffman-La Roche (Nutley, NJ). Because of retinoid photosensitivity, all experiments were performed under dim light. Samples and reference compounds were stored at −20°C or +4°C. Retinoids were dissolved at the desired concentration in ethanol. Other reagents used in the extraction process, analysis, or standard preparations were Optima grade hexane, methanol, HPLC grade water, and Tracemetal grade acetic acid.

### Cell Culture

The human cell line K562 was obtained from ATCC (Manassas, VA) and maintained in RPMI 1640 supplemented with 10 mM HEPES, 1 mM sodium pyruvate, 10% (v/v) fetal bovine serum, 1% L-glutamine and 1% penicillin-streptomycin at 37°C in an atmosphere of 5% CO_2_.

### Cellular Adhesion Assays

Static adhesion assays to immobilized ligands were adapted from established techniques [53], [54]. Briefly, fibronectin, FN-120, FN-40, or vitronectin were immobilized at desired concentrations on 96-well Immulon-2 HB microtiter plates (Thermo Scientific, Waltham, MA) in a total volume of 100 μl of 0.1 M NaHCO_3_ pH 8.4 overnight at 4°C. Nonspecific adhesion was minimized by blocking wells with 2% (w/v) bovine serum albumin (BSA) in 0.1 M NaHCO_3_ at room temperature for 1 hr. K562 cells were cultured for the designated times (24, 48, or 72 hrs) in the presence of 9-*cis*-RA (1 μM), troglitazone (1–30 μM), troglitazone and 9-*cis*-RA (15 μM and 1 μM, respectively) or an equimolar concentration of ethanol. Troglitazone was replenished every 24 hours. Before addition to wells, cells were washed twice in HEPES-Tyrodes buffer, enumerated, and added to wells (9×10^4^/well) in HEPES-Tyrodes with 1 mM MnCl_2_. When appropriate, the isotype control, MOPC-21, or the function-blocking antibody SAM-1 (anti-integrin α5) was used at final concentrations of 1, 5, or 10 μg/mL and added simultaneously to cells in HEPES-Tyrodes with MnCl_2_. Cells were incubated in the presence or absence of inhibitor for 30 min (fibronectin ligand) or 1 hr (FN-120, FN-40, or vitronectin ligands) at 37°C in 5% CO_2_. After three consecutive washes with HEPES-Tyrodes, wells were analyzed for bound cells by determining the relative cellular acid phosphatase activity within each well. Phosphatase assay buffer (1% v/v Triton X-100, 50 mM sodium acetate at pH 5.0 and 6 mg/mL *p*-nitrophenyl phosphate) was added to wells, and wells were incubated with the substrate for 30 minutes at 37°C. Color was disclosed by addition of 50 μl/well of 1 *N* NaOH. Absorbance values were obtained at 405 nm using a Biotek microplate reader. Adherent cells/well were determined from a standard curve generated using known numbers of cells. Adhesion values obtained with wells coated exclusively with BSA were considered as background values for each experimental condition and were subtracted before reporting final values.

### Extraction Procedure

K562 cells were treated with equimolar concentration of ethanol, 15 μM troglitazone, 1 μM 9-*cis-*RA or co-cultured with troglitazone and 9-*cis*-RA (15 μM and 1 μM, respectively) for 72 hrs. Troglitazone was replenished every 24 hrs. Media was harvested and stored at −20°C until extraction. Samples were thawed at room temperature. Ten milliliters of ethanol was added and the solution was mixed. The samples were acidified using 2 *N* HCl and 10 mL of hexane was immediately added. The samples were mixed and stored on ice for 20 minutes. The upper layer was removed and hexane was added and removed twice. The hexane fractions were combined and evaporated to dryness. The residual sample was stored at −20°C until HPLC analysis [55]. For all metabolism experiments, 9-*cis*-RA was added to cell-free media and allowed to incubate for 72 hrs and processed as previously described. These samples served as a control for oxidation/isomerization processes that may occur in the extraction process. Additionally, control cell-free media was spiked with 13-*cis*-RA, or 4-oxo-RA standards to verify the extraction procedure and to aid in identification of retinoid artifacts. Retinoid standards were analyzed by HPLC for purity prior to use in cell culture or retinoid artifact identification.

### HPLC Analysis

Reverse-phase HPLC analysis was performed using a Waters Model 6000A delivery system, a 6-port Rheodyne sample injector, and a Waters Millennium Chromatography Manager. The latter consists of a pump control module, a 996 photodiode array detector, and the Millennium^32^ chromatography software. Analytical separations were carried out on a stainless steel (23.5 cm×0.47 cm) Whatman Partisil 5 ODS-3 5 μm particle column. The HPLC gradient consisted initially of 50% methanol: 50% 0.01 M acetic acid which was employed for 10 minutes followed by a 60 minute linear gradient up to 100% methanol which was used to elute for 20 minutes. Flow rate was 1.00 mL/min. The retinoid absorption spectra were recorded between 200–450 nm. Chromatograms were monitored at 350 nm.

### Flow Cytometry

Cells treated with 1 μM 9-*cis*-RA, 15 μM troglitazone, 9-*cis*-RA and troglitazone (1 μM and 15 μM, respectively) or equimolar concentration of vehicle for 72 hrs were harvested and washed twice in PBS containing 3.0% (w/v) BSA (PBS-BSA). 1.0×10^6^ cells were re-suspended in a total volume of 1 mL PBS-BSA, primary anti-alpha 5-or-alpha 8 integrin antibody (10 μg/mL) was added, and cells were incubated on ice for 30 min. Cells were washed twice and FITC-conjugated secondary antibody was added at manufacturer’s recommendations, and incubated on ice for 30 min. Unbound secondary antibody was removed by two final washes in PBS-BSA, and fluorescent intensity was analyzed on Beckman-Coulter Quanta SC flow cytometer (Beckman-Coulter, Inc. Brea, CA). Cellular auto-fluorescence and non-specific secondary antibody binding was evaluated with cells lacking primary antibody. For direct flow cytometry, 1.0×10^6^ cells were re-suspended in a total volume of 1 mL PBS-BSA, anti-αvβ3-integrin antibody, anti-β1-integrin antibody, anti-β3-integrin antibody, anti-αvβ5-integrin antibody or isotype control (10 μg/mL) was added, and cells were incubated on ice for 30 min. Unbound antibody was removed by three final washes in PBS-BSA, and fluorescent intensity was analyzed.

### BrdU Cell Proliferation Assay

The ELISA-based BrdU cell proliferation kit was purchased from Chemicon International (Temecula, CA). Cells were plated at 2.5×10^4^ cells/mL in 100 μl culture media containing 1 μM 9-*cis-*RA, 15 μM troglitazone, troglitazone and 9-*cis*-RA (15 μM and 1 μM, respectively), or an equimolar concentration of ethanol and cultured for a total of 72 hrs at 37°C at 5% CO_2_ atmosphere. All treatments were diluted with cell media to desired concentrations to avoid proliferation effects due to high ethanol concentrations. BrdU reagent was added 12–24 hours prior to fixation and detection. Absorbance values were obtained with a microplate reader at 450 nm.

### Statistical Analysis

LSD was used to test for differences among groups (P = 0.01) when multiple treatments were conducted. When applicable, the Student’s *t*-test (p<0.01) was performed on treatments consisting of comparisons between control group and a single treatment.

## Results

### Troglitazone Exposure Decreases K562 Cellular Adhesion to RGD Containing Extracellular Matrix Proteins in a Concentration-dependent Manner

**Figure 1:**
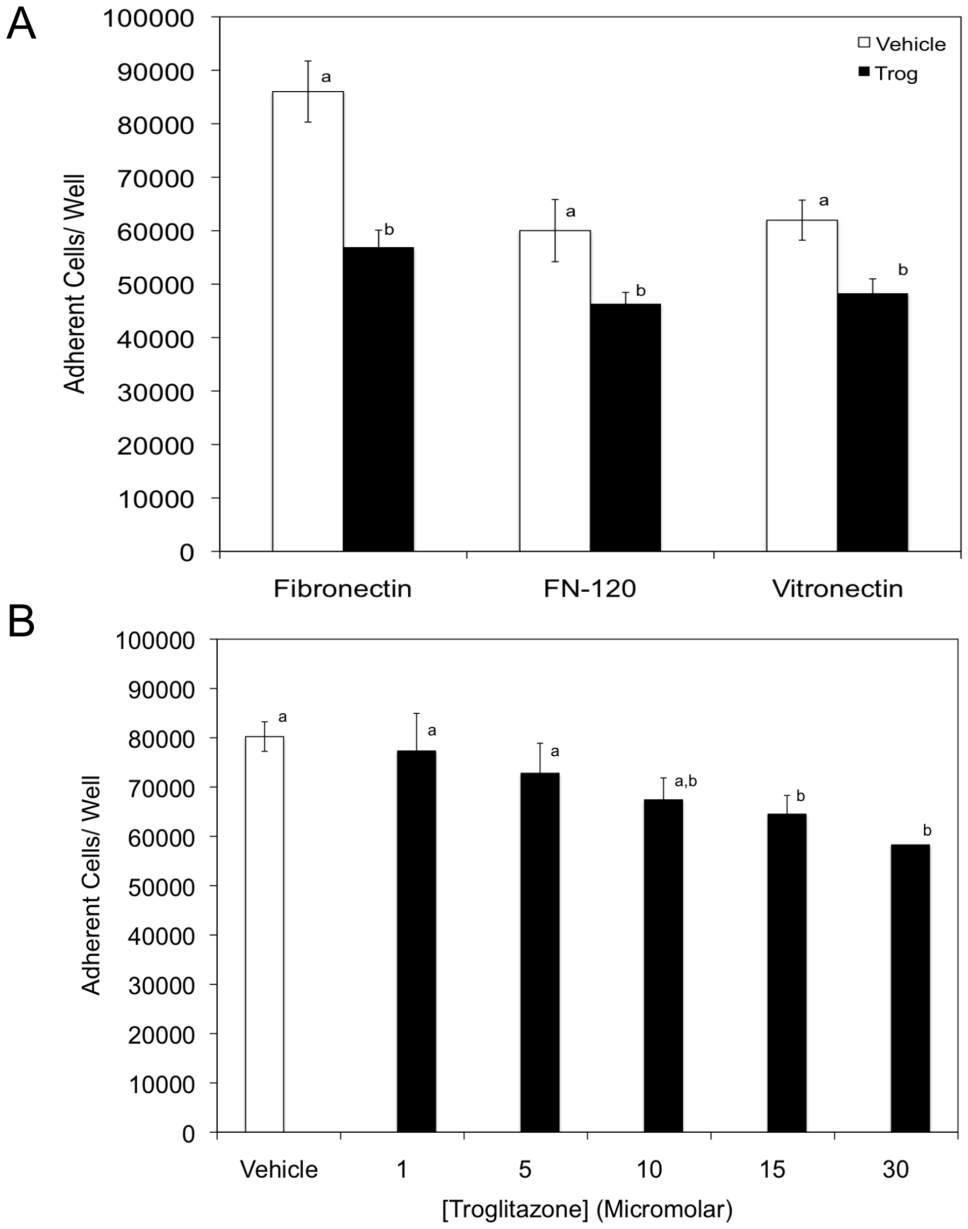
Troglitazone treatment dampens K562 cell adhesion RGD containing extracellular matrix proteins fibronectin, FN-120, and vitronectin. (A) Microtiter wells were coated with 5 μg/mL fibronectin, FN-120, or vitronectin. K562 cells cultured for 72 hrs in the presence of vehicle (*white bars*) or 15 μM troglitazone (*black bars*) were added to wells (9×10^4^ cells/well) in Hepes-Tyrodes buffer containing 1 mM MnCl_2_. (B) K562 cells cultured for 72 hrs with vehicle (*white bars*) or various concentrations of troglitazone (*black bars*) were added to wells coated with 5 μg/mL FN-120, and adhesion assays were repeated. Specific numbers of adherent cells/well were obtained by correlating experimental absorbance values to a standard curve. Adhesion values obtained with wells coated exclusively with BSA were considered as background values for each experimental condition and were subtracted before reporting final values. Adherent cells/well = adherent cells_(protein)_ – adherent cells_(BSA)_. Results are expressed as means ± SD, n = 6, Student’s t-test (p<0.01) (Figure 1A) or LSD (P = 0.01) (Figure 1B) was used to test for differences among groups. Means followed by the same letter are not significantly different.

The PPARγ agonist troglitazone has generally been considered to have an anti-inflammatory role with reduction in endothelial cell adhesion molecule MAdCAM-1 and α4β7-integrin dependent adhesion [52]. To evaluate if troglitazone alters cellular adhesion in K562 cells, we employed a static cell adhesion assay utilizing fibronectin, FN-120, or vitronectin, which were selected due to their well-defined RGD binding sites. As shown in Figure 1A, K562 cells treated with 15 μM troglitazone for 72 hours had significantly lower levels of cellular adhesion to fibronectin, FN-120, and vitronectin at 5 μg/mL as compared to vehicle. On the fibronectin ligand, troglitazone treated cells had a 37% decrease in adhesion compared to vehicle treatment. Troglitazone treated cells had a 23% reduction in cellular adhesion when compared to vehicle on FN-120 and vitronectin ligands, respectively. Interestingly, fibronectin contains binding sites for α5β1, αvβ3 and α4β7 integrins [53], [56], [57]. To determine the contribution to cellular adhesion by α4β7 integrins, the CS-1 heparin binding region fibronectin 40 kDa α chymotryptic fragment (FN-40) was utilized and cellular adhesion assays were repeated. K562 cells treated with 15 μM troglitazone or equimolar concentration of ethanol were not adhesive to this substrate (data not shown).

To evaluate whether dampened cellular adhesion was concentration dependent, K562 cells were cultured in troglitazone (1–30 μM) or equimolar concentration of ethanol. At troglitazone concentrations of 15 or 30 μM, cellular adhesion was significantly decreased compared to vehicle on the FN-120 ligand (Figure 1B). K562 cells cultured in the presence of 1 and 5 μM troglitazone exhibited no significantly different levels in cellular adhesion to FN-120 compared to vehicle. This data demonstrates that troglitazone decreases cellular adhesion to a well-defined α5 integrin ligand in a concentration dependent manner.

### 9-*cis*-Retinoic Acid Restores K562 Cellular Adhesion to Extracellular Matrix Proteins in a Time Dependent Manner

**Figure 2:**
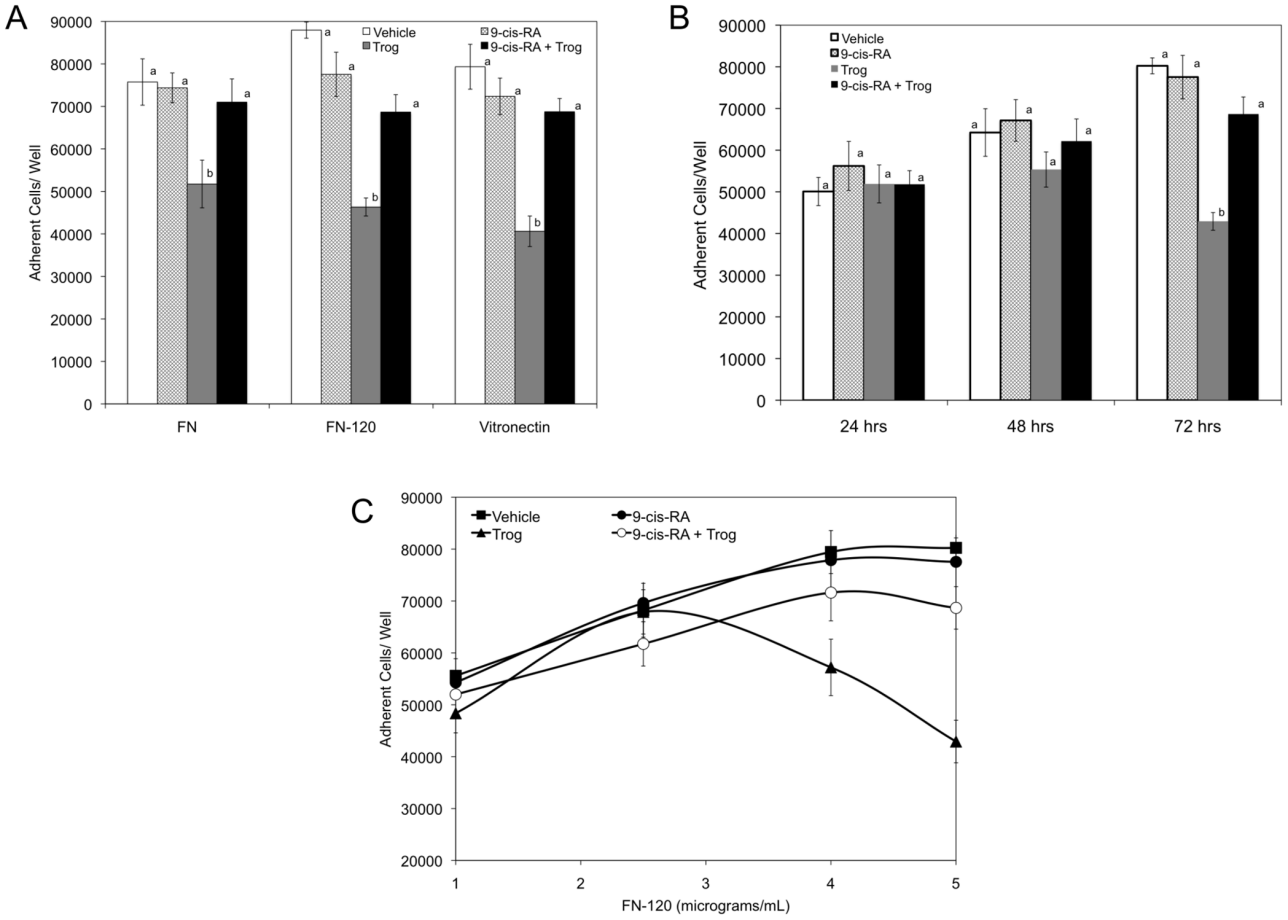
K562 cell adhesion to RGD containing extracellular matrix proteins is restored in the presence of troglitazone and 9-*cis*-RA in a concentration and time dependent manner. (A) K562 cells cultured for 72 hrs in the presence of vehicle (*white bars*), 1 μM 9-*cis*-RA (*hatched bars),* 15 μM troglitazone (*gray bars)*, or 1 μM 9-*cis*-RA and 15 μM troglitazone (*black bars)* were added to wells coated with 5 μg/mL of fibronectin, FN-120, or vitronectin. (B) K562 cells cultured for 24, 48, or 72 hrs in the presence of vehicle (*white bars*), 1 μM 9-*cis*-RA (*hatched bars),* 15 μM troglitazone (*gray bars)*, or 1 μM 9-*cis*-RA and 15 μM troglitazone (*black bars)* were added to wells coated with 5 μg/mL of FN-120. (C) Microtiter wells were coated with various concentrations of FN-120. K562 cells cultured for 72 hrs in the presence of vehicle (▪), 1 μM *9-cis-*RA (•), 15 μM troglitazone (▴), or 1 μM *9-cis-*RAand 15 μM troglitazone (○), were added to wells (9×10^4^ cells/well) in Hepes-Tyrodes buffer containing 1 mM MnCl_2_. Results are expressed as means ± SD, n = 6, LSD was used to test for differences among groups. Means followed by the same letter are not significantly different (P = 0.01).

The individual roles of PPARγ and retinoid agonists in cellular adhesion have been documented; however, their combinatory roles in this critical facet of immune cell function are not well established. Previously, retinoids have been shown to augment cellular adhesion [54]. To evaluate if 9-*cis*-RA impacts cellular adhesion, K562 cells were cultured in the presence of 1 μM 9-*cis*-RA, 15 μM troglitazone, equimolar concentration of ethanol, or co-cultured in 9-*cis*-RA and troglitazone (1 μM and 15 μM, respectively) and static cellular adhesion assays were repeated with fibronectin, FN-120, FN-40, or vitronectin. In the presence of 9-*cis*-RA, cellular adhesion levels were comparable to vehicle levels for fibronectin, FN-120, and vitronectin ligands (Figure 2A). Treatment with troglitazone resulted in a marked decrease in cellular adhesion to fibronectin, FN-120, and vitronectin substrates. Interestingly, statistically higher levels of adhesion were achieved for cells co-cultured with troglitazone and 9-*cis*-retinoic acid for 72 hrs on fibronectin, FN-120, and vitronectin substrates with adhesion levels comparable to vehicle control. For all treatment groups, K562 cell adhesion was not observed on the FN-40 substrate (data not shown).

To address if the observed augmented adhesion was time-dependent, we restricted the exposure of K562 cells to these agonists for 24, 48, or 72 hrs. When cells were added to wells coated with FN-120, similar levels of cellular adhesion were obtained for all treatment groups cultured for 24 hours (Figure 2B). At 48 hours, a slight decrease in cellular adhesion was observed in K562 cells treated with troglitazone followed by negligible restoration in cellular adhesion in the presence of troglitazone and 9-*cis*-RA. However, there were no statistical differences between cellular adhesion levels of K562 cells treated with vehicle, 9-*cis*-RA, troglitazone, or 9-*cis*-RA and troglitazone for 48 hours. At 72 hours, statistically lower cellular adhesion levels were obtained in troglitazone treated cells with a decrease of 47% in cellular adhesion compared to vehicle treatment only. K562 cells co-cultured with troglitazone and 9-*cis*-RA for 72 hours had comparable levels of cellular adhesion to vehicle treatment alone. Our data demonstrates that treatment with troglitazone results in a decrease in cellular adhesion that is time dependent. Further, in the presence of troglitazone, 9-*cis*-RA restores cellular adhesion levels to that of vehicle treatment alone within a 72 hour time period.

Since 9-*cis*-RA restores cellular adhesion in the presence of troglitazone to FN-120 at 5 μg/mL, we evaluated whether the combinatory treatments of troglitazone and 9-*cis*-RA confers K562 cell adhesion to FN-120 in does dependent and saturable manner. As shown in Figure 2C, saturation curves between vehicle and 9-*cis*-RA treated cells were comparable. K562 cells cultured in the presence of 15 μM troglitazone had continual decreasing levels of adhesion to FN-120 at concentrations exceeding 2.5 μg/mL. In contrast, at FN-120 concentrations greater that 2.5 μg/mL, cells co-cultured with 9-*cis*-RA and troglitazone demonstrated a marked increase in cellular adhesion compared to troglitazone treatment alone with the combinatory treatment groups producing greater than 30% percent more adhesion when compared to troglitazone treated cells.

### Troglitazone Alters the Bioavailability of 9-cis-RA in K562 Cells

**Figure 3:**
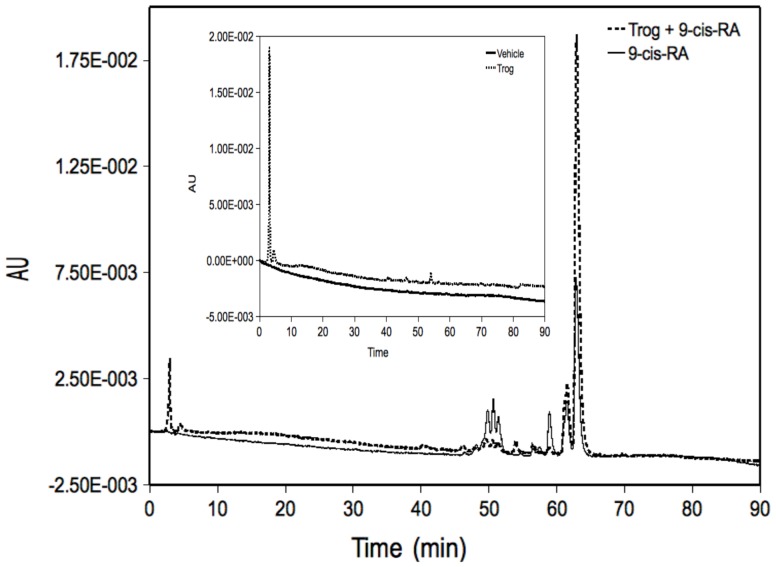
9-*cis*-retinoic acid metabolism in K562 cells is altered in the presence of troglitazone. K562 cells were cultured for 72 μM 9-*cis*-RA, 15 μM troglitazone, or 1 μM 9-*cis*-RA and 15 μM troglitazone. Cells and media were collected, pooled, and extracted as previously described, and HPLC analysis was performed. Chromatograms are shown with 9-*cis*-RA media extract (*black line*) or 9-*cis*-RA and troglitazone media extract (*dashed line*). Chromatograms of the control vehicle media extract (*black line*) and troglitazone media extract (*dashed line*) are shown in the inset.

Ultimately, the genetic regulation elicited by retinoids is dictated by ligand availability, which is influenced by retinoid metabolism. Through retinoid profiling, we have previously demonstrated that all-*trans*-retinoic acid is not metabolized in a number of lymphocyte cell lineages [54]. Additionally, we have metabolically profiled 9-*cis*-RA in number of cell lines including RPMI 8866, Jurkat, and Daudi (Figures S1, S2, and S3, respectively). However, the metabolism of 9-*cis*-RA in K562 cells has not been conducted. Given the ability of troglitazone to inhibit key cytochrome P450 isozymes involved in the oxidative metabolism of 9-*cis*-RA, it is paramount to determine if troglitazone impacts retinoid metabolism in this cell line. K562 cells were cultured in the presence of vehicle, 1 μM 9-*cis*-RA, 15 μM troglitazone or 9-*cis*-RA and troglitazone (1 μM and 15 μM, respectively) for 72 hours. Media volumes were normalized among treatment groups to account for cell proliferation changes. HPLC profiles of the 72-hour K562 media extracts are shown in Figure 3. For 9-*cis*-RA media extracts, the parent compound was detected at 63 minutes with a maximum absorbance at 348.5 nm. 13-*cis*-retinoic acid was identified at 62 minutes with a maximum absorbance of 353.2 nm. Several polar metabolites were detected between 49–52 minutes. The CYP_450_-dependent metabolite, 4-oxo-retinoic acid, was detected in K562 9-*cis*-RA media extract at 50 minutes and maximum absorbance of 362 nm. When troglitazone and 9-*cis*-RA were metabolically profiled in K562 cells, an increase in peak height of the parent compound, 9-*cis*-RA, was observed at 62 minutes. Additionally, metabolites observed in the 49–52 minute retention time period were diminished with 4-oxo-retinoic acid not detected in samples treated with troglitazone and 9-*cis*-RA. To control for artifact production, cell-free media containing 9-*cis*-RA was incubated for 72 hours and the extraction was repeated. Chromatographic profiles of cell-free treatments were comparable to the 9-*cis*-RA standard (data not shown). To validate extraction and chromatographic procedures for oxidative metabolism, cell-free media was spiked with 4-oxo-retinoic acid and the extraction procedure was repeated. The HPLC chromatograms were comparable to the standard 4-oxo-retinoic acid with a retention time 50 minutes and maximum absorbance of 362 nm (data not shown). Our data demonstrates that troglitazone increases the availability of 9-*cis*-retinoic acid in K562 cells.

### Troglitazone Dampens Alpha 5 Integrin-dependent Cell Adhesion in K562 Cells

**Figure 4:**
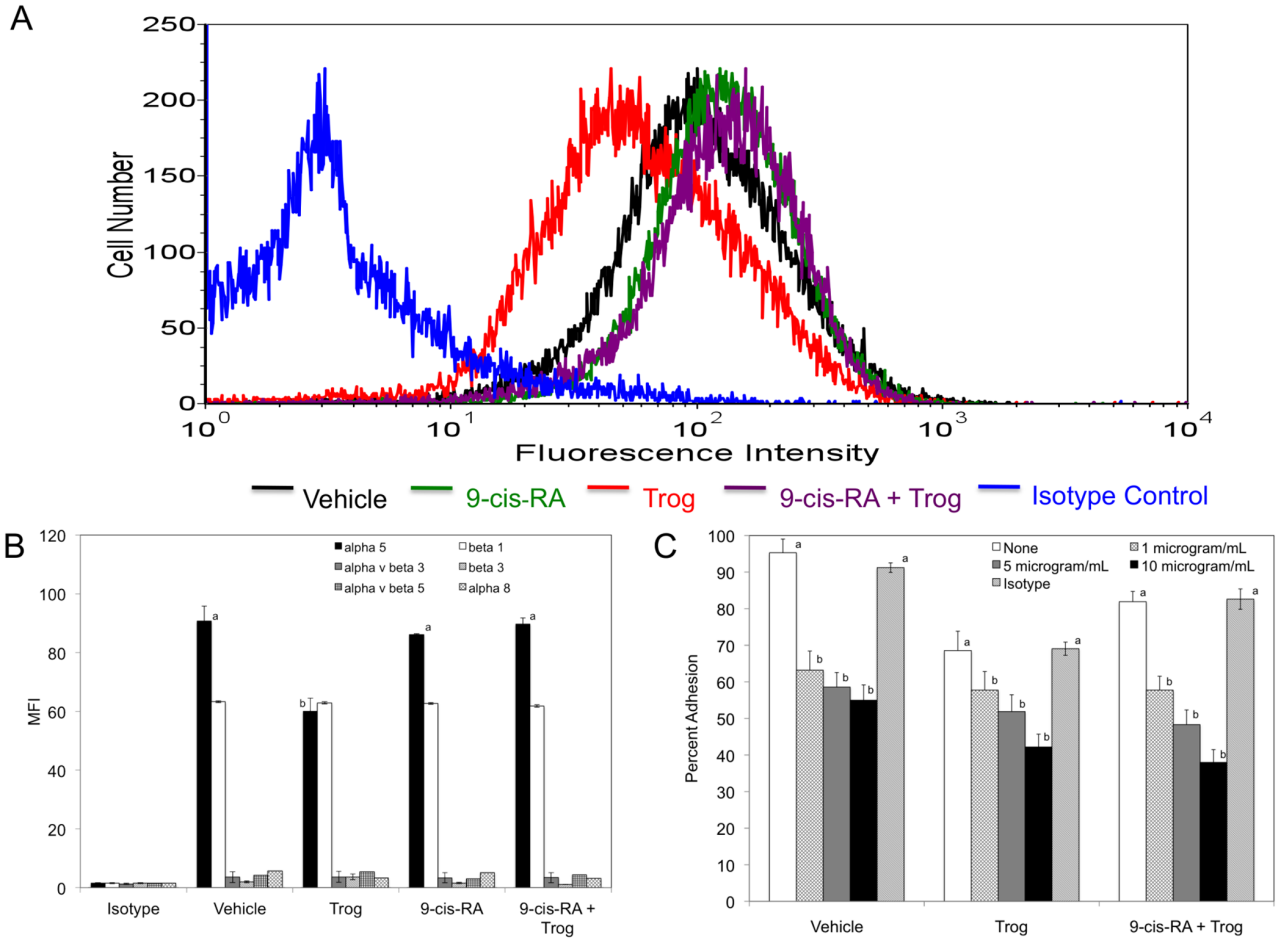
Troglitazone decreases α5 integrin-dependent cell adhesion in K562 cells. (A) The extent of mAb SAM-1 (anti-α5) binding to K562 cells exposed to vehicle, 9-*cis*-RA, troglitazone, or 9-*cis*-RA and troglitazone for 72 hrs was assessed by flow cytometric analysis. SAM-1 was used at a concentration of 10 μg/mL. Cell surface levels of the α5 integrin subunit obtained with vehicle cells (*black histogram*), 9-*cis*-RA (*green histogram*), troglitazone (*red histogram*), 9-*cis*-RA and troglitazone (*purple histogram*), or an antibody isotype control (*blue histogram*) are shown. (B) K562 cells were treated with vehicle, 9-*cis*-RA, troglitazone, or 9-*cis*-RA and troglitazone for 72 hrs and subjected to FACS analysis. The mean fluorescent intensities (MFI) of integrin expression levels are shown for alpha 5 (*black bars*), beta 1 (*white bars*), alpha v beta 3 (*gray bars*), beta 3 (*diagonal bars*), alpha v beta 5 (*small grid bars*), and alpha 8 (*hatched bars*). Results are expressed as means ± SD, n = 3, LSD was used to test for differences among groups. Means followed by the same letter are not significantly different (P = 0.01). No statistical differences were observed in MFI values for αvβ3, β1, β3, αvβ5, or α8 integrins. (C) K562 cells cultured for 72 hours in the vehicle, 1 μM 9-*cis*-RA, 15 μM troglitazone, or 1 μM 9-*cis*-RA and 15 μM troglitazone were added to wells coated with 5 μg/mL of FN-120 in the presence of none (*white bars*), 1 (*hatched bars*), 5 (*gray bars*), 10 (*black bars*) μg/mL of SAM-1, or 10 μg/mL of isotype control (*diagonal bars*). Percent adhesion = ((adherent cells_(FN-120)_ – adherent cells_(BSA)_)/input cells) X 100. Results are expressed as means ± SD, n = 6, LSD was used to test for differences among groups. Means followed by the same letter are not significantly different (P = 0.01).

Separately retinoids and thiazolidinediones have been shown to impact the integrin repertoire expressed on the cell surface [47], [48], [49], [50], [51], [58], [59]. Due to the novel adhesion response exhibited by K562 cells on fibronectin, FN-120 and vitronectin, we examined integrin expression including α5, β1, β3, αvβ3, αvβ5 and α8 when K562 cells were exposed to 1 μM 9-*cis*-RA, 15 μM troglitazone, 9-*cis*-RA and troglitazone (1 μM and 15 μM, respectively) or equimolar concentration of vehicle. Interestingly, our data demonstrates a decrease of the α5 integrin subunit expression on the cell surface when K562 cells were exposed to troglitazone for 72 hours (Figure 4A). In the presence of 9-*cis*-RA and troglitazone, cell surface α5 integrin subunit levels were comparable to vehicle treatment alone. As shown in Figure 4B, cell surface beta 1 integrins were detected in K562 cells; however, there was no significant difference in β1 subunit expression on K562 cells exposed to 1 μM 9-*cis*-RA, 15 μM troglitazone, 9-*cis*-RA and troglitazone (1 μM and 15 μM, respectively) or equimolar concentration of vehicle. Since fibronectin and vitronectin contain the binding sites for α8β1 and αvβ3 integrins, we profiled α8, β3 and αvβ3 integrins on the cell surface of K562 cells treated with vehicle, troglitazone, 9-*cis*-RA, or 9-*cis*-RA and troglitazone [60]. Although the beta 3-subunit has been detected at low levels in K562 cells, our results demonstrate that cell surface β3 subunit levels remain low in K562 cells and are not increased in the presence of troglitazone or 9-*cis*-retinoic acid and troglitazone [61]. We observe a similar trend when cell surface levels of αvβ3 and α8 were investigated (Figure 4B). Further, we examined the cell surface levels of αvβ5 since vitronectin has been shown to serve as a substrate for this integrin subclass [60]. Our data reflect very low surface levels of αvβ5 regardless of treatment group. Interestingly, Sun et. al report that alpha 5 integrins were recruited into adhesion sites induced by vitronectin in vascular smooth muscle cells [62]. Further, Pasqualini et al have shown that the alpha 5-subunit was involved in adhesion to fibronectin and vitronectin in K562 cells with fibronectin being the preferred ligand [63]. Our results are consistent with this study. Our results in Figure 2A show a robust cellular adhesion response to fibronectin when K562 cells were allowed to adhere for 30 minutes. However, when FN-120 or vitronectin were utilized as substrates, the reported cellular adhesion was observed when cells were allowed to adhere for 1 hour.

Recently, retinoids have been shown to promote cellular adhesion through integrin dependent and independent mechanisms depending on cell type [54], [64]. To determine if the restored cellular adhesion observed in the presence of 9-*cis*-RA and troglitazone was integrin-dependent, we assessed K562 cell adhesion to immobilized FN-120 in the presence of various concentrations of a function-blocking antibody to the integrin α5 subunit or isotype control. As shown in Figure 4C, K562 cells treated with troglitazone alone had a significant decrease in cellular adhesion at all integrin inhibitor concentrations tested. In the presence of 9-*cis*-RA and troglitazone, a similar decrease in cellular adhesion was observed when cells were added to wells coated with FN-120. Interestingly, when cells were incubated in the absence of 1 mM Mn^2+^, an exogenous integrin activator, cellular adhesion to FN-120 was not observed regardless of treatment group (data not shown). Collectively, our data suggests that in the presence of troglitazone, 9-*cis*-RA impacts K562 cellular adhesion by integrin-dependent mechanisms.

### Troglitazone and 9-cis-Retinoic Acid Synergistically Decreases Cellular Proliferation in K562 Cells

**Figure 5:**
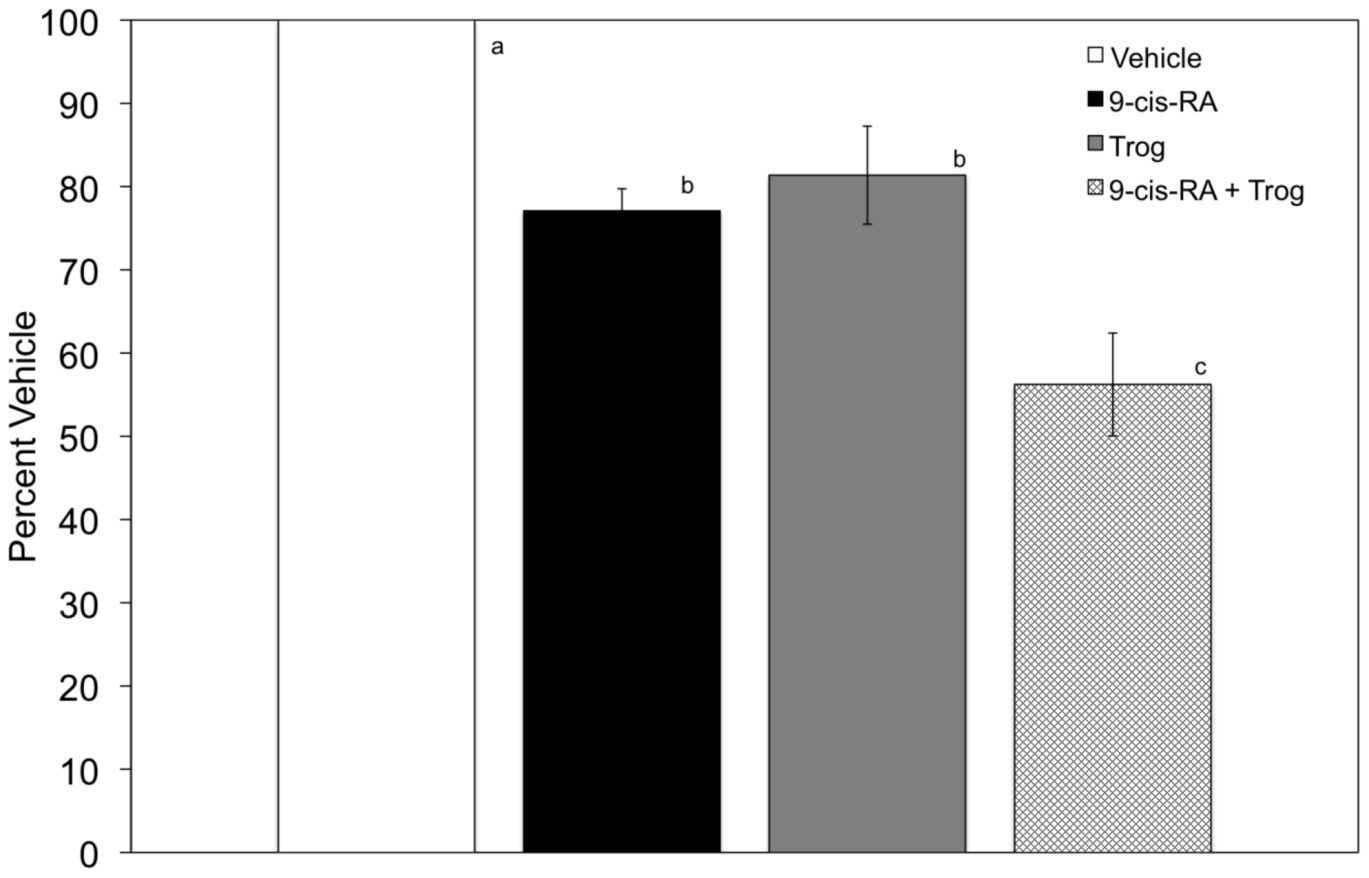
K562 cellular proliferation is decreased in the presence of troglitazone and 9-*cis*-RA. Cells were cultured with vehicle (*white bar*), 1 μM 9-*cis-*RA (*black bar*), 15 μM troglitazone (*gray bar*), or 15 μM and 1 μM troglitazone and 9-*cis*-RA (*hatched bar*), respectively, for 72 hrs. BrdU incorporation was assessed by ELISA and proliferation values were normalized using Percent Vehicle = (Abs_(treatment)_/Abs_(vehicle)_) X 100. Results are shown as means ± SD, n = 6, LSD was used to test for differences among groups. Means followed by the same letter are not significantly different (P = 0.01).

The balance between cellular adhesion and proliferation is fundamental to mounting an effective immune response. Cells require anchorage to extracellular matrix proteins to proliferate. Due to the novel cellular adhesion response elicited by the combinatory treatment of 9-*cis*-RA and troglitazone, we assessed their impact on cellular proliferation. K562 cells were cultured in the presence of vehicle, troglitazone, 9-*cis*-RA, or the combinatory treatment of 9-*cis*-RA and troglitazone for 72 hours and proliferation was quantitated as shown in Figure 5. In the presence of 9-*cis*-RA, significantly lower levels of cellular proliferation were observed when compared to vehicle. A similar trend was observed when cells were treated with troglitazone. Proliferation levels were comparable with a 20% decrease in proliferation in cells treated with troglitazone or 9-*cis*-RA only. Interestingly, the combined treatment of 9-*cis*-RA and troglitazone resulted in a 44% decrease in proliferation levels when compared to vehicle alone.

## Discussion

The current study focuses on the combinatory effects of 9-*cis*-RA and troglitazone on cellular adhesion and proliferation in the human erythroleukemia cell line, K562. We present evidence that troglitazone dampens cellular adhesion to RGD containing extracellular matrix proteins fibronectin, FN-120, and vitronectin, but adhesion to FN-40, which contains the binding site for α4β7 integrins, was not observed. Recently, K562 cells have been reported to be 9-*cis*-RA non-responsive with respect to cellular adhesion on α4 integrin counter receptors [64]. However, our data support that in the presence of troglitazone, 9-*cis*-RA restores K562 cellular adhesion to RGD containing extracellular matrix proteins that are established α5 integrin ligands. Although troglitazone has been shown to reduce α4β7 dependent lymphocyte adhesion, K562 cells have not been reported to contain the α4β7 integrin, and α5β1 integrin has been demonstrated to be the major integrin expressed in K562 cells with low level of expression of beta 3 integrins also found [61], [65]. We have profiled other integrins including αvβ3 and α8β1, which can adhere to fibronectin. In our study, the cell surface levels of αvβ3 and α8 were low in all treatments tested. We assessed the contribution of the integrin β1 subunit to the cellular adhesion changes observed in the presence of troglitazone and 9-cis-RA and troglitazone, and our results demonstrate that the β1 subunit cell surface levels are not changed upon treatment with these agonists. Further, our data demonstrates that troglitazone dampens cellular adhesion in a concentration dependent manner with concentrations greater than 10 μM significantly decreasing cellular adhesion to FN-120. The troglitazone concentrations utilized in our study are near physiological concentrations reported [66].

Retinoids and thiazolidinediones have been independently investigated with respect to their role in cellular adhesion. Due to the promiscuous nature of RXR and its ability to partner with PPARγ, our study examined the combinatory effects of 9-*cis*-RA and troglitazone on cellular adhesion. We present evidence that 9-*cis*-RA restores cellular adhesion to RGD containing extracellular matrix proteins in the presence of troglitazone. The time-dependent cellular adhesion restoration elicited by retinoid exposure may be due to alterations in ligand availability and/or receptor pairing. Cellular retinoid availability and sequestration influence heterodimer/homodimer partnerships, which control transcription of retinoid responsive genes. We demonstrate that troglitazone increases the bioavailability of 9-*cis*-RA, which could allow for transcription of target genes that are involved in the restored cellular adhesion observed at 72 hours. Additionally, cell signaling may play a critical role in retinoid receptor pairing. For example, one of the paramount responses in inflammation is the activation of NF-κB, which consists of a p65 and p50 heterodimers. Phosphorylation of the p65 subunit allows for activation of NF-κB and entry into the nucleus where NF-κB directly interacts with the DNA binding domain of RXRα [67]. However, troglitazone inhibits phosphorylation of the p65 subunit of NF-κB thus allowing RXRα to function as a homodimer or as a heterodimeric partner with other receptors, such as PPARγ [52]. We suggest that the observed restoration in cellular adhesion in the presence of both 9-*cis*-RA and troglitazone at 72 hours is likely to be a combination of not only increased retinoid availability, but also the complex transcriptional regulation that is involved in promiscuous retinoid receptor pairings. Alternatively, the changes in cell surface α5 subunit may be attributed to differential recycling to/from the membrane. Integrin heterodimer endocytosis can follow more than one internalization route. For example, α5β1 integrin internalization has been demonstrated to occur through clathrin-dependent, caveolin-dependent, and clathrin-independent mechanisms [68].

Due to the complicated cell signaling pathways that retinoids and integrins elicit, it is plausible to suggest that there may be a number of signaling factors that may contribute to retinoid dependent restoration of cellular adhesion to vitronectin. We demonstrate that cellular adhesion to vitronectin is decreased in the presence of troglitazone and restored in the presence of troglitazone and 9-*cis*-RA. Other studies have shown that alpha 5 integrins are capable of interacting with vitronectin; however, αvβ5, αvβ3 and α8β1 integrins have been clearly established as vitronectin receptors [60], [62], [63]. Recently, there have been some very interesting investigations with regard to urokinase-type plasminogen activator receptor (uPAR), which acts as high affinity receptor for vitronectin. The vitronectin-uPAR complex is capable of activating intracellular signaling through partnering with integrins, including α5β1 [69], [70], [71]. Further, PPARγ agonists have been show to decrease uPAR expression in leukocytes, while uPAR is induced in the presence of retinoic acid in certain cell types [72], [73]. Interestingly, this may explain some of the cellular adhesion effects that we observe with these agonists. Our data demonstrates a decrease in cell surface α5 subunit expression in the presence of troglitazone and a decrease in cellular adhesion to vitronectin. We speculate that that troglitazone may be decreasing uPAR and/or α5 integrin levels thus decreasing attachment to vitronectin through the uPAR-α5β1 complex. In the presence of the troglitazone and 9-*cis*-RA, uPAR and/or α5 integrin levels may be increased, and cellular adhesion is restored to vitronectin. A model proposed by Tarui et al. lends support to our speculation [70]. Alternatively, the observed adhesion to vitronectin could be mediated by αIIbβ3 or may be integrin independent.

The role of integrins in cell differentiation is multifaceted involving changes in integrin repertoire and interactions with extracellular matrix proteins. Retinoids and PPARγ agonists have been separately demonstrated to alter integrin expression [58], [74]. The K562 cell line is a distinctive cell line, which can be stimulated into differentiating into erythroid or monocytic-macrophage cell lines. Cell surface integrin alterations have been reported in erythroid and monocyte differentiation [75]. For example, hemin-stimulated erythroid differentiation was accelerated in K562 cells when cells adhered to fibronectin through their interaction with α5β1 [76]. Additional studies show that monocyte differentiation is dependent on the binding of α5β1 to the RGD domain of fibronectin [77]. Clearly, the interactions between fibronectin and α5β1 integrins play a considerable role in differentiation. We provide evidence that the combinatory treatment of troglitazone and 9-*cis*-RA acid restores cellular adhesion to RGD containing extracellular matrix proteins through interactions with the α5 integrin subunit. We speculate that these agonists through their involvement with alpha 5 integrins may effect cellular differentiation. The observed α5 integrin subunit cell surface induction could be a result from a direct pairing of PPARγ-RXRα. Alternatively, an increase in retinoid availability could transcriptionally modulate integrins and/or extracellular matrix proteins expression patterns. Further, cellular maturity may be influenced by the combination of retinoid exposure and integrin repertoire. In cells capable of differentiation, such as K562, NB-4, and HuT-78, oxidative metabolism of retinoids occurs [78], [79]. NB-4 and HuT-78 cells are retinoid responsive with respect to cellular adhesion on α4 integrin substrates; however, cellular adhesion to α5 integrin ligands has not been investigated in the presence of retinoids [64]. In more mature cells, such as Jurkat, Daudi, and RPMI 8866 retinoids are not metabolized (see Figure S1, Figure S2, and Figure S3, respectively) [54]. Interestingly, Jurkat cells are retinoid non-responsive with respect to cellular adhesion on α4 integrin ligands; however, cellular adhesion to α5 integrin substrates has not been conducted in the presence of retinoids. Daudi and RPMI 8866 cells have the α4 integrin subclass on the cell surface, while the α5 integrin subclass has not been reported. RPMI 8866 cells have retinoid inducible integrin-independent cellular adhesion to α4 integrin ligands while Daudi cells are not retinoid responsive with respect to adhesion [54]. Clearly, the impact of retinoid availability on α5 integrin dependent cellular adhesion and differentiation warrants further investigation.

Retinoids are well established in their ability to regulate proliferation in many cell types and impact cell adhesion. Currently, the contributions of retinoids and thiazolidinediones in cell adhesion and proliferation have been independently examined. Within the past decade, there have been limited studies examining the role of both agonists in cellular proliferation. Based upon the current study, it is clear that in the presence of troglitazone K562 cells are retinoid responsive with respect to cellular adhesion and proliferation. We demonstrate that the combination of these agonists synergistically decrease cellular proliferation. Further, we suggest that this observed decrease in proliferation might be due to the ability of these agonists to increase cell surface α5 integrin subunit expression. Integrins are vital players in cell survival by providing adhesive interactions that trigger signals that direct cell cycle progression. Interestingly, in the absence of fibronectin, α5β1 integrins have been shown to activate a signaling pathway that leads to a decrease in cellular proliferation [80]. We suggest that these agonists modulate α5 integrin expression and that α5 integrins may be a focal point in the potential partnership between cellular proliferation and adhesion.

Cellular adhesion, proliferation, and differentiation are critical events in maintaining cell homeostasis, particularly for immune cells. The current study provides insight into the role of retinoids and thiazolidinediones in K562 cellular proliferation and adhesion. Our study is the first to demonstrate that the combinatory treatment of the PPAR agonist, troglitazone, and the RXR agonist, 9-*cis*-RA, results in augmented cellular adhesion, altered retinoid metabolism, restored cell surface α5 integrin expression, and dampened cellular proliferation in a human erythromyeblastoid leukemia cell line. To shed light on how these agonists impact cellular adhesion and proliferation, future studies will need to characterize the specific hetero-or homodimeric receptor partnerships involved in cellular adhesion and proliferation, identify the specific cellular signals that are responsible for agonist dependent restored cellular adhesion, and determine the contribution of integrins to cellular proliferation and adhesion in the presence of these agonists.

## Supporting Information

Figure S1 **9-*cis*-retinoic acid metabolism in the mature B-cell line RPMI 8866.** RPMI 8866 cells were cultured for 72 hrs in the presence of vehicle (ethanol) or 1 μM 9*-cis*-RA. Chromatograms are shown with vehicle treated media extract (*blue line*) and 9-*cis*-RA treated media (*red line*). 9-*cis-*RA was detected at 63.57 minutes with a maximum absorbance at 348.5 nm. The absorption spectrum of 9-*cis*-RA is shown in the insert.
(TIFF)

Figure S2 **9-*cis*-retinoic acid metabolism in Jurkat human T-lymphoblastoma cells.** Jurkat cells cultured for 72 hrs in the presence of vehicle (ethanol) or 1 μM 9-*cis*-RA. Cells and media were collected, pooled, and extracted as previously described. Chromatograms are shown with vehicle treated media extract (*blue line*) and 9-*cis*-RA treated media (*red line*). 9-*cis*-RA had a maximum absorption of 348.5 nm with a retention time of 63.29 minutes. The absorption spectrum for 9-*cis*-RA is shown in the insert.
(TIFF)

Figure S3 **9-*cis*-retinoic acid metabolism in Daudi B-cells.** Daudi cells cultured for 72 hrs in the presence of vehicle (ethanol) or 1 μM 9-*cis*-RA. Chromatograms are shown with vehicle treated media extract (*blue line*) and 9-*cis*-RA treated media (*red line*). 9-*cis*-RA had a maximum absorption of 348.5 nm with a retention time of 62.88 minutes. The absorption spectrum for 9-*cis*-RA is shown in the insert.
(TIFF)
