# Supplementary figures and images for: 9-cis-Retinoic Acid and Troglitazone Impacts Cellular Adhesion, Proliferation, and Integrin Expression in K562 Cells
Source: PLoS One. 2014 Mar 26;9(3):e93005. doi: 10.1371/journal.pone.0093005 (PMC3966848; doi:10.1371/journal.pone.0093005)

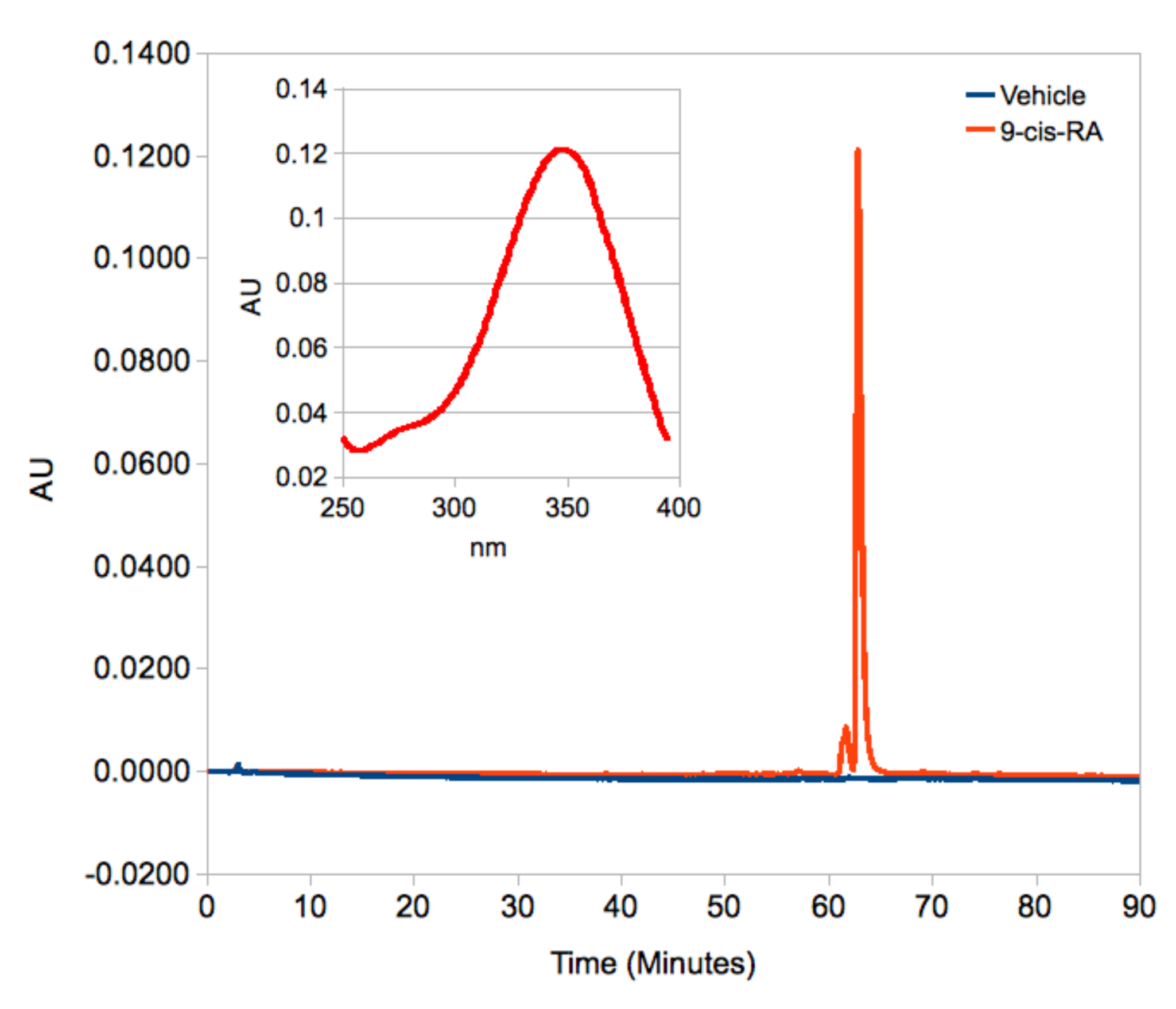

Supplement: Figure S1 — 9-cis-retinoic acid metabolism in the mature B-cell line RPMI 8866. RPMI 8866 cells were cultured for 72 hrs in the presence of vehicle (ethanol) or 1 μM 9-cis-RA. Chromatograms are shown with vehicle treated media extract (blue line) and 9-cis-RA treated media (red line). 9-cis-RA was detected at 63.57 minutes with a maximum absorbance at 348.5 nm. The absorption spectrum of 9-cis-RA is shown in the insert. (TIFF) [file pone.0093005.s001.tif]

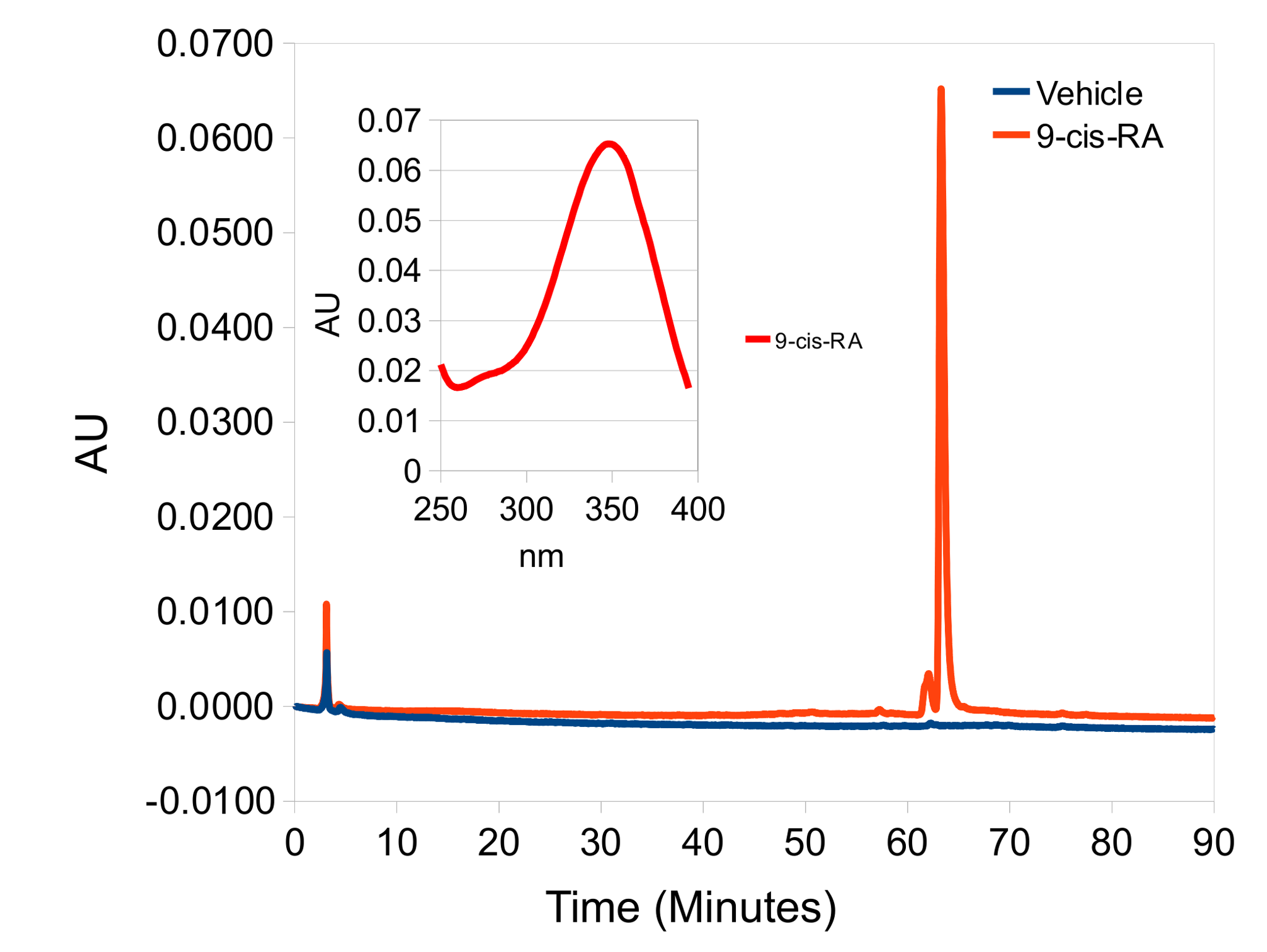

Supplement: Figure S2 — 9-cis-retinoic acid metabolism in Jurkat human T-lymphoblastoma cells. Jurkat cells cultured for 72 hrs in the presence of vehicle (ethanol) or 1 μM 9-cis-RA. Cells and media were collected, pooled, and extracted as previously described. Chromatograms are shown with vehicle treated media extract (blue line) and 9-cis-RA treated media (red line). 9-cis-RA had a maximum absorption of 348.5 nm with a retention time of 63.29 minutes. The absorption spectrum for 9-cis-RA is shown in the insert. (TIFF) [file pone.0093005.s002.tif]

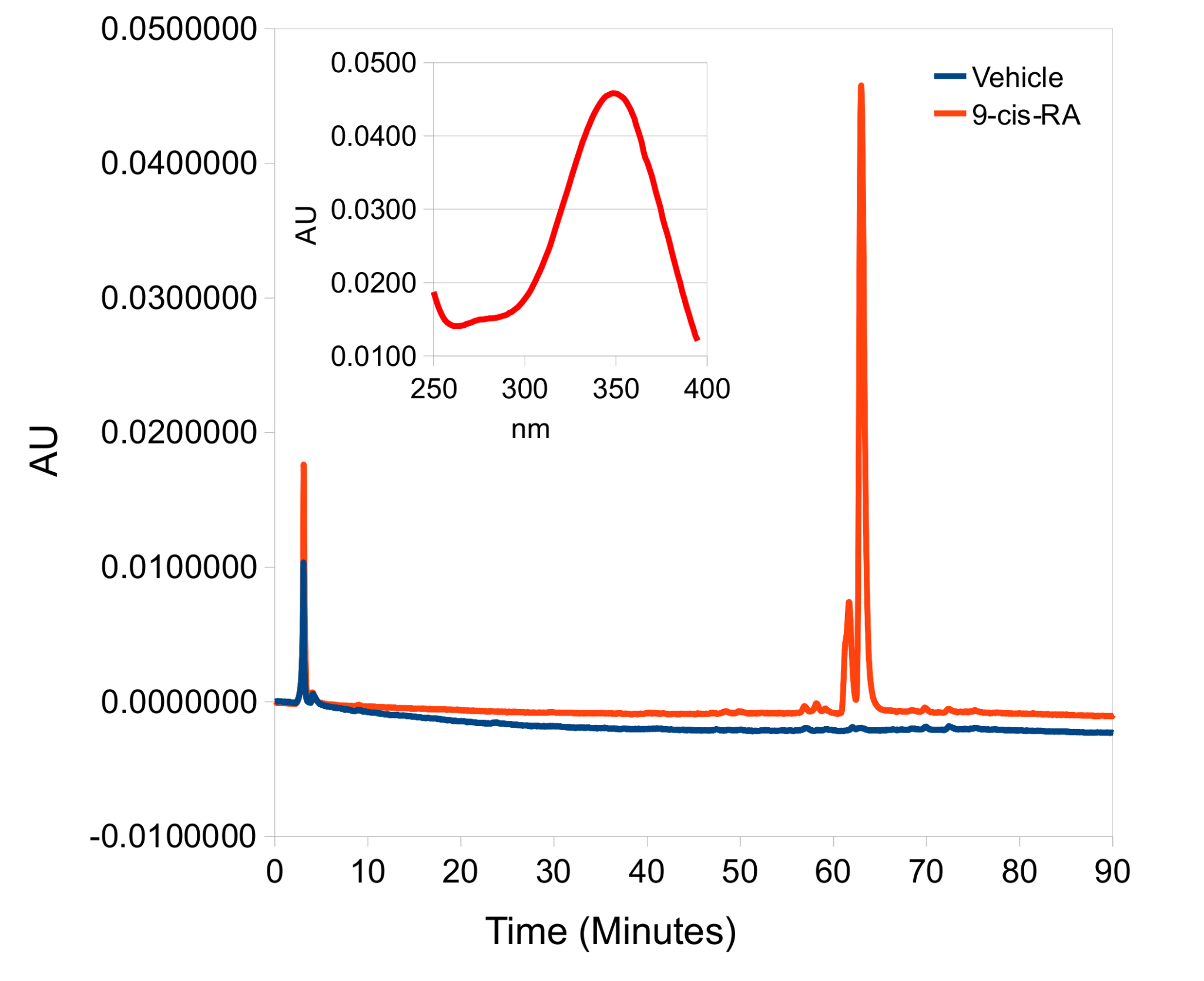

Supplement: Figure S3 — 9-cis-retinoic acid metabolism in Daudi B-cells. Daudi cells cultured for 72 hrs in the presence of vehicle (ethanol) or 1 μM 9-cis-RA. Chromatograms are shown with vehicle treated media extract (blue line) and 9-cis-RA treated media (red line). 9-cis-RA had a maximum absorption of 348.5 nm with a retention time of 62.88 minutes. The absorption spectrum for 9-cis-RA is shown in the insert. (TIFF) [file pone.0093005.s003.tif]
